# Analysis of the Relationships between Multiple Endocrine Hormones and Return of Spontaneous Circulation (ROSC) in Cardiac Arrest Patients: Possible Association of the Serum Free T4 Level with ROSC

**DOI:** 10.1155/2020/4168420

**Published:** 2020-11-30

**Authors:** Go Koizumi, Kentaro Mikura, Tatsuya Iida, Mariko Kaji, Mai Hashizume, Norimitsu Murai, Yasuyoshi Kigawa, Kei Endo, Toru Iizaka, Ryo Saiki, Fumiko Otsuka, Jun Sasaki, Munetaka Hayashi, Shoichiro Nagasaka

**Affiliations:** ^1^Division of Diabetes, Metabolism and Endocrinology, Showa University Fujigaoka Hospital, 1-30 Fujigaoka, Aoba-ku, Yokohama, Kanagawa 227-8501, Japan; ^2^Department of Critical Care and Emergency Medicine, Showa University Fujigaoka Hospital, 1-30 Fujigaoka, Aoba-ku, Yokohama, Kanagawa 227-8501, Japan

## Abstract

**Background:**

Endocrine hormones are closely associated with homeostasis, so it is important to clarify hormone secretion dynamics in shock. Few reports, however, have examined the dynamics of endogenous hormone secretion relative to prognosis in cardiac arrest patients. Therefore, to clarify the roles of endocrine hormones in out-of-hospital cardiac arrest (OHCA) patients, the concentrations of anterior pituitary, thyroid, and adrenocortical hormones were measured, and their associations with return of spontaneous circulation (ROSC) were examined.

**Methods:**

The subjects were OHCA patients transported to our Emergency Department. In addition to conventional clinical laboratory tests, the following were measured: serum TSH, serum free T3, serum free T4 (F-T4), plasma ACTH, serum cortisol, serum GH, serum IGF-1, plasma aldosterone concentration (PAC), and plasma renin activity. The primary endpoint was the presence or absence of ROSC, and the secondary endpoint was 24-hour survival.

**Results:**

A total of 29 patients, 17 in the ROSC group and 12 in the non-ROSC group, were studied. There were associations between ROSC and low serum potassium, high F-T4, low cortisol, and low PAC on bivariate analyses. There were associations between ROSC and serum potassium, F-T4, and GH using the step-wise method. On multiple logistic regression analysis, a relationship between ROSC and high serum F-T4 level was identified by both methods. There were also associations between 24-hour survival and both low serum potassium and elevated blood glucose levels.

**Conclusions:**

The present findings suggest a possible relationship between the serum F-T4 level and ROSC in OHCA patients. A higher serum F-T4 level might cause an increase in the *β*-adrenergic response in cardiomyocytes and increased responsiveness to catecholamines and was possibly associated with ROSC.

## 1. Introduction

Adrenaline is currently administered to improve cardiac arrest patient outcomes in countries around the world. In a randomized, controlled trial (RCT) involving out-of-hospital cardiac arrest (OHCA) patients, adrenaline administration was associated with improved short-term survival, but it may be detrimental to survival to hospital discharge and survival with a good neurological outcome [1, 2]. In another RCT conducted in the United Kingdom, it was reported that, although adrenaline increased the 30-day survival rate, it had an adverse effect on neurological outcome [3]. Tsai et al. reported that, in a nonrandomized, open-label, clinical study in which hydrocortisone or placebo was administered to endogenous OHCA patients, although an improvement in the return of spontaneous circulation (ROSC) rate was found in the group treated with hydrocortisone, no significant differences were found in survival or hospital discharge rates [4]. Overall, there is no medication with sufficient evidence to show improved outcomes in cardiac arrest patients.

Endocrine hormones are closely related to homeostasis, such as blood pressure, body fluids and electrolytes, and blood glucose levels, and it is important to clarify the dynamics of hormone secretion during shock. There have been reports investigating the circulating levels of cortisol and catecholamines in cardiac arrest patients and their prognosis. These reports stated that the circulating levels of adrenaline/noradrenaline were significantly lower in the ROSC group [5] and that no difference in serum cortisol levels was found between ROSC and non-ROSC groups [6]. In addition, there have been reports that low levels of plasma triiodothyronine (T3) and thyroxine (T4) were associated with a poor prognosis in cardiac arrest patients [7, 8]. However, there have been few reports of any associations between multiple endogenous hormone concentrations and cardiac arrest patient prognosis. The present study was undertaken to identify the roles of multiple endocrine hormones in OHCA patients. For this purpose, the concentrations of anterior pituitary, thyroid, and adrenocortical hormones were measured, and their relationships with ROSC were examined.

## 2. Materials and Methods

The subjects were consecutive OHCA patients, who had been found to fall and had undergone cardiopulmonary resuscitation (CPR) by a bystander, transported to the Showa University Fujigaoka Hospital Emergency Department from July to September 2014, and for whom a close relative (or legal representative) provided informed consent. CPR had been optimally performed. Patients with known endocrine disorders or who were being treated with glucocorticoid were excluded from the study, but those with comorbidities such as diabetes mellitus and hypertension were included. This study was conducted with the approval of Showa University Fujigaoka Hospital Institutional Review Board (Approval no. 2014023).

A blood sample was collected on arrival and in addition to general clinical laboratory tests such as blood glucose, hematological and biochemical examinations, and blood gases; the following were measured at the laboratory of Hoken Kagaku, Inc. (https://hokenkagaku.wixsite.com/corporateinfo/): serum thyroid-stimulating hormone (TSH, by chemiluminescent enzyme immunoassay (CLEIA)), serum free T3 (F-T3, by CLEIA), serum free T4 (F-T4, by CLEIA), plasma adrenocorticotropic hormone (ACTH, by electrochemiluminescence immunoassay), serum cortisol (by chemiluminescence immunoassay), serum growth hormone (GH, by CLEIA), serum insulin-like growth factor-1 (IGF-1, by radioimmunoassay (RIA)), plasma aldosterone concentration (PAC, by RIA), and plasma renin activity (PRA, by RIA). Normal reference ranges for these tests are shown in Table 1. The interassay and intraassay variations for TSH, F-T3, and F-T4 were less than 3.1% and 2.4%, respectively.

After arrival, advanced cardiovascular life support (ACLS), such as chest compression, airway management, and administration of adrenaline, was performed under the American Heart Association Guidelines [9]. ROSC was defined by feeling the pulsation of the carotid or brachial arteries after cardiopulmonary resuscitation [10].

Using the presence or absence of ROSC as the primary endpoint and 24-hour survival as the secondary endpoint, their relationships with the general clinical laboratory test results and hormone concentrations were examined. The results are expressed as median values and range (minimum-maximum). Analyses of the various parameters were performed either by the chi-squared test (sex) or logistic regression analysis relative to the presence or absence of ROSC, and multiple logistic regression analysis was then performed for significant associations. Similarly, factors related to ROSC were selected by the step-wise method, and multiple logistic regression analysis was then performed for significant associations. The Wald test was used to test the odds ratios and 95% confidence intervals resulting from multiple logistic regression analysis. Discrimination analyses were also performed for each analysis mentioned above. Analyses of the various parameters were performed either by the chi-squared test (sex) or logistic regression analysis relative to the presence or absence of 24-hour survival. Statistical analysis was performed using JMP Pro version 14.0.0 (SAS Institute Inc., Cary, NC, USA). The level of significance was set at *p* < 0.05.

## 3. Results

A total of 29 patients were involved in the study. Their ages ranged from 41 to 93 years; there were 17 men and 12 women, 17 were in the ROSC group and 12 in the non-ROSC group (Table 1). No significant differences were found between the ROSC group and the non-ROSC group in sex, age, body temperature, blood gases, lactic acid, serum sodium, white blood cell count, and blood glucose, but the serum potassium level was significantly lower in the ROSC group (*p* = 0.0061). No significant differences were found in serum TSH, serum F-T3, plasma ACTH, serum GH, serum IGF-1, and PRA, but the serum F-T4 level was significantly higher in the ROSC group (*p* = 0.0491), and both serum cortisol (*p* = 0.0424) and PAC (*p* = 0.0290) levels were significantly lower in the ROSC group. These 4 variables were then designated ROSC target variables, and multiple logistic regression analysis was performed. It was found that F-T4 had the highest odds ratio (315.5), and F-T4 was identified as a significant factor by the likelihood ratio test (*p* = 0.0150, Table 2).

There were associations between ROSC and serum potassium, F-T4, and GH using the step-wise method. On multiple logistic regression analysis, potassium and F-T4 were identified as significant factors by the likelihood ratio test (*p* = 0.0013 and *p* = 0.0023, Table 2). GH was considered nonsignificant, since the 95% confidence interval contained 1 by the Wald test (Table 3).

Discrimination analyses showed the percentage of correct classifications and goodness of fit (*R*^2^) to be 88.0% and 0.44719 (Table 2) and 84.6% and 0.51041 (Table 3), respectively. In both analyses, the percentage of correct classifications and goodness of fit (*R*^2^) were considered to have good properties, i.e., the former >75% and the latter >0.3.

The outcome for 14 of the 17 patients in the ROSC group was recorded as in-hospital death because there was no hope for recovery and aggressive treatment was waived. The remaining 3 patients were transferred to another facility for rehabilitation. There were 7 patients, including the 3 recorded as survived to hospital discharge, for the secondary endpoint of 24-hour survival after transport.

Within the ROSC group (*n* = 17), when the 24-hour survival group (*n* = 7) was compared with the nonsurvival group (*n* = 10) (Table 4), the serum potassium level was significantly lower (*p* = 0.0052), and the blood glucose was significantly higher (*p* = 0.0120) in the 24-hour survival group, but no significant differences were found in other markers.

**Figure 1:**
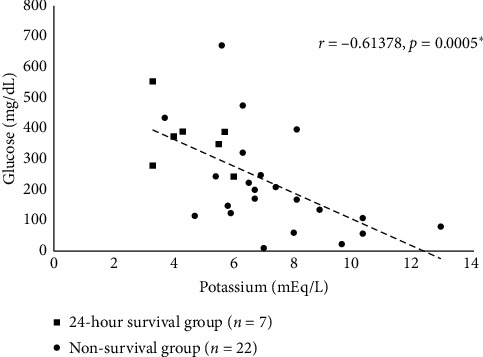
Correlation between the serum potassium and blood glucose levels (*n* = 29).

In the comparison of the 24-hour survival group (*n* = 7) and the nonsurvival group (*n* = 22) in all patients (*n* = 29) (Table 5), the serum potassium level was significantly lower (*p* = 0.0004), and the blood glucose was significantly higher (*p* = 0.0312) in the 24-hour survival group, but no differences were found in other markers. In these patients, an inverse correlation was found between the serum potassium and blood glucose levels (*r* = −0.61378, *p* = 0.0005) (Figure 1).

## 4. Discussion

In the investigation of the 29 OHCA patients in the present study, correlations were found between ROSC and low serum potassium, high serum F-T4, low serum cortisol, and low aldosterone levels, and on multiple logistic regression analysis, a relationship between ROSC and a high serum F-T4 level was identified. Similarly, using the step-wise method and the subsequent multiple logistic regression analysis, a relationship between ROSC and the serum F-T4 level was confirmed, and discrimination analyses confirmed the association. There were also associations of low serum potassium and high blood glucose levels with 24-hour survival.

Regarding the factors relevant to ROSC, Lin et al. reported that hyperkalemia was associated with bradycardia, nonsinus rhythm, oliguria, and acidosis and that the duration of survival was shorter in patients presenting with hyperkalemia [11]. Shida et al. reported that, with hyperkalemia, neurological outcomes deteriorated significantly in a concentration-dependent manner and that the serum potassium level was an effective prognostic indicator for achieving ROSC [12]. In the present study, both ROSC and 24-hour survival outcomes were poor in patients presenting with hyperkalemia.

Regarding serum cortisol levels in cardiac arrest patients, Miller et al. reported that the serum cortisol levels were high in resuscitated patients and that relative adrenal insufficiency (low serum cortisol) in response to stress was associated with a poor prognosis [13]. They postulated that vascular reactivity and function modulated by glucocorticoid might be impaired due to relative adrenal insufficiency. However, it has been reported elsewhere that, as in the present study, higher serum cortisol levels were associated with worse outcomes [6, 14] so that this matter remains controversial. As an explanation for the association between higher serum cortisol levels and poor outcomes, Mosaddegh et al. suggested that serum cortisol levels would be higher before cardiac arrest due to chronic disease [6]. In fact, in the present study, there was a positive correlation (*p* = 0.0361) between plasma ACTH and serum cortisol levels in the ROSC group alone, but not in all patients. The present findings suggest that high serum cortisol levels in the non-ROSC group may not reflect an acute stress response.

Very few studies concerning an association between PAC and either cardiac arrest or ROSC have been reported. In the analysis of all patients in the present study, there was a positive correlation (*p* = 0.0005) between PAC and PRA. A high PRA means a decrease in either renal blood flow or perfusion pressure, and it is possible that clinical conditions associated with high PRA and high PAC levels were present before cardiac arrest so that the PAC level could have been high in the non-ROSC group.

Regarding thyroid hormones and shock, Borkowski et al. reported that low serum F-T3 and TSH levels in septic shock were significant prognostic factors for mortality [15]. Longstreth et al. reported their retrospective study of cardiac arrest patients, in which low levels of plasma T3 and T4 and a high level of plasma TSH were associated with a poor prognosis [7]. Wortsman et al. reported that patients immediately after cardiac arrest had lower serum T3, T4, and F-T4 levels than severely ill noncardiac arrest patients [8]. The finding of the present study that a higher serum F-T4 level is associated with ROSC does not contradict these reports.

Type 2 deiodinase is expressed in cardiac muscle and regulates the T3 concentration in cardiomyocytes [16]. Type 2 deiodinase promotes the conversion from T4 to T3 in cells and regulates intracellular T3 concentrations [17]. In animal models treated with levothyroxine (LT4), dosing with LT4 increased the density of *β*-adrenergic receptors in heart ventricles [18, 19]. Carvalho-Bianco et al. reported that treatment with LT4 increased *β*-adrenergic responsiveness in cardiomyocytes mediated by a change in the expression of G proteins in membrane complexes [20]. Moreover, Kuzman et al. showed that administration of LT4 resulted in an increase in heart weight and left ventricular systolic pressure mediated by activation of the Akt signal transduction pathway in cardiomyocytes [21]. In the present study, high serum F-T4 levels were associated with ROSC. It is possible that high serum F-T4 levels in cardiac arrest patients may have caused an increase in *β*-adrenergic responsiveness in cardiomyocytes similar to the administration of LT4, which increased the responsiveness to catecholamines, and were thereby associated with ROSC. In fact, adrenaline was administered to all patients as a resuscitation measure in the present study.

There was also an association between 24-hour survival and low serum potassium levels. As noted above, it has been reported that postresuscitation cardiac function and hemodynamics deteriorated with hyperkalemia and that the duration of survival was shorter in patients presenting with hyperkalemia [11]. In the present study, there was also an association between 24-hour survival and higher blood glucose levels. A high blood glucose level under stress results from an increase in insulin resistance [22]. Cardiac arrest patients are considered to be under high stress and to have higher blood glucose levels than their usual levels. Patients who do not develop hyperglycemia under such circumstances may have other intrinsic factors leading to a poor prognosis, such as poor nutrition. In fact, in the present study, there was a positive correlation (*r* = 0.5470, *p* = 0.0231) between the blood glucose level and the serum cholinesterase level, which is an indicator of nutritional status. Because this was a study of cardiac arrest patients, it was not possible to determine body mass index or other nutritional indicators accurately.

In the present study, there was a strong inverse correlation between the serum potassium and blood glucose levels (Figure 1). It is conceivable that cortisol, renin-aldosterone, and insulin secretion may have been involved in this relationship, but there were no correlations between either the serum potassium or blood glucose level and serum cortisol, plasma ACTH, PAC, and PRA levels (data not shown). Insulin levels were not measured in the present study. Vihonen et al. previously reported that circulating insulin and glucagon levels showed a positive correlation with blood glucose levels in patients after cardiac arrest [23]. In the present study, the serum potassium level was higher and the blood glucose level tended to be lower in the non-ROSC group, but further studies that include measurements of insulin and related factors are needed.

The limitations of the present study included that the number of patients was small; clearly, a study involving a larger number of patients is needed. Because it was a cross-sectional study, it remains unclear whether a true causal relationship between ROSC and serum F-T4 levels exists. It is also unclear whether a chronic, latent elevation in the serum F-T4 level contributes to ROSC, or whether an acute rise in the F-T4 level is involved. It seems important to measure endogenous hormone levels, especially F-T4, consecutively in survivors, but this could not be done in the present study. In addition, background factors that could not be evaluated in the present study, such as duration of cardiopulmonary arrest time, may also be related to ROSC and the survival rate. Zwemer et al. conducted a study of continuous administration of LT4 before and after cardiac arrest in dogs and reported that LT4 administration significantly increased cardiac output and increased systemic oxygen supply and consumption, and it offered neuroprotective benefits compared to the control group [24]. In addition, D'Alecy reported that T3 and rT3 administration failed to improve neurologic outcomes [25]. However, it is still unclear whether such interventions can be justified in humans.

## 5. Conclusions

In the present study, relationships between multiple endocrine hormones and ROSC in OHCA patients were analyzed, and a possible association between the serum F-T4 level and ROSC was identified. More such studies, especially larger studies, would increase knowledge and provide suggestions for future therapeutic interventions to improve the rate of ROSC and intact survival for OHCA patients.

## Figures and Tables

**Table 1:** Comparison between the ROSC and non-ROSC groups.

	ROSC group (*n* = 17)	Non-ROSC group (*n* = 12)	*p* value
Sex (male/female)	12/5	5/7	0.1194
Age (y)	83 (52–92)	78 (41–93)	0.4077
Temperature (°C)	37.2 (32.0–38.4)	34.6 (32.0–38.0)	0.9087
Arterial blood gas (pH) [7.35–7.45]	6.954 (6.685–7.405)	6.824 (6.415–7.418)	0.1943
Arterial blood PO_2_ (mmHg) [80–100]	78.2 (33.8–157)	92.8 (37.4–167)	0.1694
Arterial blood PCO_2_ (mmHg) [35–45]	58.1 (10.0–337)	29.1 (9.1–288)	0.4263
Arterial blood bicarbonate (mEq/L) [21–27]	18.4 (8.4–36.5)	17.9 (8.5–33.5)	0.8407
Lactic acid (mmol/L) [0.5–2.0]	10.6 (5.89–18.0)	13.6 (1.67–23.4)	0.3085
Na (mEq/L) [135–147]	142 (134–147)	146 (134–164)	0.2202
K (mEq/L) [3.6–5.0]	5.8 (3.3–8.1)	6.9 (4.7–12.9)	0.0061^*∗*^
White blood cell count (/*μ*L) [3500–9100]	10500 (5000–17100)	11350 (3800–17800)	0.9860
Blood glucose (mg/dL) [70–109]	248 (60–554)	135 (23–671)	0.2630
TSH (*μ*IU/mL) [0.390–4.010]	2.211 (0.296–7.954)	3.292 (0.260–45.89)	0.2230
F-T3 (pg/mL) [2.2–4.1]	2.56 (1.99–3.64)	2.40 (1.60–4.64)	0.5874
F-T4 (ng/dL) [0.83–1.71]	1.00 (0.64–1.63)	0.91 (0.56–1.06)	0.0491^*∗*^
ACTH (pg/mL) [7.2–63.3]	25.4 (2.7–269)	31.9 (4.7–152)	0.8283
Cortisol (*μ*g/dL) [4.5–21.1]	13.2 (4.3–41.3)	27.6 (2.5–74.1)	0.0424^*∗*^
GH (ng/mL)	4.04 (0.37–24.5)	4.79 (0.16–11.7)	0.5849
IGF-1 (ng/mL)	74 (15.0–146)	39.3 (8.4–188)	0.4206
Aldosterone (pg/mL) [35.7–240]	135 (49.3–260)	186 (49.6–3038)	0.0290^*∗*^
PRA (ng/mL/h) [0.2–2.7]	2.3 (0.4–31.9)	2.4 (0.3–69.4)	0.2153

Data are shown as medians (range), except sex. Normal reference ranges are given in square brackets. *p* values were determined by logistic regression analysis, except for sex (chi-squared test). *∗p* < 0.05. ROSC, return of spontaneous circulation; Na, sodium; K, potassium; TSH, thyroid stimulating hormone; F-T3, free T3; F-T4, free T4; ACTH, adrenocorticotrophic hormone; GH, growth hormone; IGF-1, insulin-like growth factor-1; PRA, plasma renin activity.

**Table 2:** Multiple logistic regression analysis for ROSC using K, F-T4, cortisol, and aldosterone levels.

	Likelihood ratio test, *p* value	Odds ratio	Range
K (mEq/L)	0.1268	0.6103	0.2584–1.1397
F-T4 (ng/dL)	0.0150^*∗*^	315.51	2.5538–432311
Cortisol (*μ*g/dL)	0.4229	0.9618	0.8462–1.0710
Aldosterone (pg/mL)	0.1829	0.9982	0.9807–1.0005

Regression equation, *p* = 0.0040; *∗p* < 0.05. ROSC, return of spontaneous circulation; K, potassium; F-T4, free T4.

**Table 3:** Multiple logistic regression analysis for ROSC using K, F-T4, and growth hormone levels.

	Likelihood ratio test, *p* value	Odds ratio	Range
K (mEq/L)	0.0013*∗*	0.3208	0.1200–0.8572
F-T4 (ng/dL)	0.0023*∗*	1732.9	2.1465–1398992
GH (ng/mL)	0.0162	1.3261	0.9852–1.7848

Regression equation, *p* = 0.0004; *∗p* < 0.05. ROSC, return of spontaneous circulation; K, potassium; F-T4, free T4; GH, growth hormone.

**Table 4:** Comparison of the 24-hour survival and nonsurvival groups in the ROSC group (*n* = 17).

	24-hour survival group (*n* = 7)	Nonsurvival group (*n* = 10)	*p* value
Sex (male/female)	6/1	6/4	0.3382
Age (y)	85 (52–87)	80 (64–92)	0.8913
Temperature (°C)	35.0 (34.3–38.4)	34.8 (32.0–36.0)	0.0941
Arterial blood gas (pH)	6.912 (6.685–7.090)	6.977 (6.758–7.405)	0.2386
Arterial blood PO_2_ (mmHg)	85.1 (42.9–157)	69.9 (33.8–124)	0.1182
Arterial blood PCO_2_ (mmHg)	72.3 (27.2–179)	57.9 (10.0–337)	0.8605
Arterial blood bicarbonate (mEq/L)	18.4 (12.7–23.4)	16.3 (8.4–36.5)	0.9395
Lactic acid (mEq/L)	12.6 (8.92–14.2)	10.5 (5.89–18.0)	0.7404
Na (mEq/L)	140 (135–146)	143 (134–147)	0.7609
K (mEq/L)	4.3 (3.3–6.0)	6.8 (3.7–8.1)	0.0052^*∗*^
White blood cell count (/*μ*L)	10500 (8200–17000)	9500 (5000–17100)	0.4094
Blood glucose (mg/dL)	374 (243–554)	205 (60–435)	0.0120^*∗*^
TSH (*μ*IU/mL)	1.950 (0.306–5.160)	2.472 (0.296–7.954)	0.2877
F-T3 (pg/mL)	2.56 (2.10–3.56)	2.56 (1.99–3.64)	0.8359
F-T4 (ng/dL)	1.03 (0.90–1.43)	0.97 (0.64–1.63)	0.6803
ACTH (pg/mL)	28.3 (3.2–269)	22.7 (2.7–132)	0.3578
Cortisol (*μ*g/dL)	9.0 (4.3–30.2)	15.0 (6.5–41.3)	0.2147
GH (ng/mL)	2.41 (1.51–7.74)	5.69 (0.37–24.5)	0.0726
IGF-1 (ng/mL)	90.3 (32.0–146)	71.1 (15.0–110)	0.3011
Aldosterone (pg/mL)	162 (49.3–223)	130 (58.8–260)	0.9151
PRA (ng/mL/h)	3.5 (1.1–6.0)	2.0 (0.4–32)	0.2025

Data are shown as medians (range), except sex. *p* values were determined by logistic regression analysis, except for sex (chi-squared test). *∗p* < 0.05. ROSC, return of spontaneous circulation; Na, sodium; K, potassium; TSH, thyroid stimulating hormone; F-T3, free T3; F-T4, free T4; ACTH, adrenocorticotrophic hormone; GH, growth hormone; IGF-1, insulin-like growth factor-1; PRA, plasma renin activity.

**Table 5:** Comparison of the 24-hour survival and nonsurvival groups in all patients (*n* = 29).

	24-hour survival group (*n* = 7)	Nonsurvival group (*n* = 22)	*p* value
Sex (male/female)	6/1	11/11	0.1872
Age (y)	85 (52–87)	78 (41–93)	0.6134
Temperature (°C)	35.0 (34.3–38.4)	34.8 (32.0–38.0)	0.1900
Arterial blood gas (pH)	6.912 (6.685–7.090)	6.903 (6.415–7.418)	0.8496
Arterial blood PO_2_ (mmHg)	85.1 (42.9–157)	83.6 (33.8–167)	0.5703
Arterial blood PCO_2_ (mmHg)	72.3 (27.2–179)	40.2 (9.1–337)	0.8336
Arterial blood bicarbonate (mEq/L)	18.4 (12.7–23.4)	17.8 (8.4–36.5)	0.8749
Lactic acid (mEq/L)	12.6 (8.92–14.2)	11.4 (1.67–23.4)	0.7687
Na (mEq/L)	140 (135–146)	143 (134–164)	0.4347
K (mEq/L)	4.3 (3.3–6.0)	6.8 (3.7–13)	0.0004^*∗*^
White blood cell count (/*μ*L)	10500 (8200–17000)	13500 (3800–17800)	0.4595
Blood glucose (mg/dL)	374 (243–554)	171 (23–671)	0.0312^*∗*^
TSH (*μ*IU/mL)	1.950 (0.306–5.160)	2.882 (0.260–45.89)	0.2233
F-T3 (pg/mL)	2.56 (2.10–3.56)	2.45 (1.60–4.64)	0.6990
F-T4 (ng/dL)	1.03 (0.90–1.43)	0.95 (0.56–1.63)	0.1663
ACTH (pg/mL)	28.3 (3.2–269)	23.2 (2.7–152)	0.4574
Cortisol (*μ*g/dL)	9.0 (4.3–30.2)	21.2 (2.5–74.1)	0.0546
GH (ng/mL)	2.41 (1.51–7.74)	4.79 (0.16–24.5)	0.1143
IGF-1 (ng/mL)	90.3 (32.0–146)	62.0 (8.4–188)	0.2927
Aldosterone (pg/mL)	162 (49.3–223)	152 (49.6–3038)	0.2237
PRA (ng/mL/h)	3.5 (1.1–6.0)	2.3 (0.3–69)	0.1157

Data are shown as medians (range), except sex. *p* values were determined by logistic regression analysis, except for sex (chi-squared test). ^*∗*^*p* < 0.05. ROSC, return of spontaneous circulation; Na, sodium; K, potassium; TSH, thyroid stimulating hormone; F-T3, free T3; F-T4, free T4; ACTH, adrenocorticotrophic hormone; GH, growth hormone; IGF-1, insulin-like growth factor-1; PRA, plasma renin activity.

## Data Availability

The data used in this research are anonymized and stored by the data administrator. Requests for data should be addressed to the author.
